# Effect of Regulatory Element DNA Methylation on Tissue-Type Plasminogen Activator Gene Expression

**DOI:** 10.1371/journal.pone.0167588

**Published:** 2016-12-14

**Authors:** Sylvie Dunoyer-Geindre, Anne-Sophie Rivier-Cordey, Carlos Caetano, Richard J. Fish, Egbert K. O. Kruithof

**Affiliations:** 1 Division of Angiology and Hemostasis, University Medical Center, University of Geneva, Geneva, Switzerland; 2 Department of Genetic Medicine and Development, University Medical Center, University of Geneva, Geneva, Switzerland; Beijing Cancer Hospital, CHINA

## Abstract

Expression of the tissue-type plasminogen activator gene (t-PA; gene name *PLAT*) is regulated, in part, by epigenetic mechanisms. We investigated the relationship between *PLAT* methylation and *PLAT* expression in five primary human cell types and six transformed cell lines. CpG methylation was analyzed in the proximal *PLAT* gene promoter and near the multihormone responsive enhancer (MHRE) -7.3 kilobase pairs upstream of the *PLAT* transcriptional start site (TSS, -7.3 kb). In Bowes melanoma cells, the *PLAT* promoter and the MHRE were fully unmethylated and t-PA secretion was extremely high. In other cell types the region from -647 to -366 was fully methylated, whereas an unmethylated stretch of DNA from -121 to +94 was required but not sufficient for detectable t-PA mRNA and t-PA secretion. DNA methylation near the MHRE was not correlated with t-PA secretion. Specific methylation of the *PLAT* promoter region -151 to +151, inserted into a firefly luciferase reporter gene, abolished reporter gene activity. The region -121 to + 94 contains two well-described regulatory elements, a PMA-responsive element (CRE) near -106 and a GC-rich region containing an Sp1 binding site near +59. Methylation of double-stranded DNA oligonucleotides containing the CRE or the GC-rich region had little or no effect on transcription factor binding. Methylated CpGs may attract co-repressor complexes that contain histone deacetylases (HDAC). However, reporter gene activity of methylated plasmids was not restored by the HDAC inhibitor trichostatin. In conclusion, efficient *PLAT* gene expression requires a short stretch of unmethylated CpG sites in the proximal promoter.

## Introduction

Tissue-type plasminogen activator (t-PA) produced by vascular endothelial cells plays an important role in the removal of intravascular fibrin deposits [1,2]. In the brain, t-PA, produced by neurons, astrocytes, glial cells and cerebral EC, contributes to synaptic plasticity, learning, long term potentiation and neuronal cell migration, but may also contribute to pathological events, such as glutamate-mediated excitotoxicity, demyelination, cerebral inflammation, Alzheimer’s disease, seizures and disruption of the blood brain barrier [3–10]. Besides EC and brain cells, t-PA is produced by many other cell types, including smooth muscle cells, fibroblasts, keratinocytes, peritoneal mesothelial cells, cardiac myocytes and gingival cells [11]. The large variety of cell types producing t-PA, as well as the diversity of drugs, hormones, cytokines and growth factors that modulate *PLAT* expression in these cells, suggests that t-PA has additional, still ill-defined, functions outside the vascular and central nervous systems [11].

Some information is available on promoter/enhancer elements regulating agonist-mediated changes in *PLAT* expression in different cell types. The proximal *PLAT* promoter contains an AP1- and CRE-binding site and several GC-rich sites that bind members of the Sp1 family [12–15] and a multihormone responsive enhancer (MHRE) is located at –7.3 kb [16,17]. Histone deacetylases (HDAC) are known to suppress t-PA production in EC. Indeed, HDAC inhibitors such as trichostatin or valproic acid strongly increase t-PA expression by EC [18–20]. The effect of HDAC inhibition appears to be direct because it is correlated with changes in histone acetylation at the *PLAT* promoter [18–20]. This implies that epigenetic mechanisms suppress t-PA production in EC. In a porcine ischemia model, treatment with valproic acid increased t-PA secretion twofold in response to a transient coronary occlusion [21]. A study of *PLAT* promoter methylation in human umbilical vein EC (HUVEC) and in primary human hepatocytes and hepatoma cells suggested that promoter methylation is associated with a low production of t-PA [19]. An unmethylated proximal *PLAT* promoter was also observed in human astrocytes and human neurons and postmortem human brain tissue [22]. A recent study by Magnusson et al. [23] observed that culturing human endothelial cells led to a demethylation of the MHRE, which was associated with an increase in t-PA expression.

In view of the important role of t-PA in the vascular system and in the brain, as well as its potential role in other tissues, it is important to better understand the relation between epigenetic mechanisms and cell-type specific *PLAT* expression. In the present study we tried to identify sites that contribute to the inhibitory effect of DNA methylation. We concentrated our studies on the proximal promoter of the *PLAT* gene and on the MHRE and investigated the association of the methylation state of these regions with the degree of t-PA secretion in human primary cell types (EC, monocytes, fibroblasts, hepatocytes and astrocytes) and six transformed cells lines. Our results suggest that CpG methylation of the proximal *PLAT* gene promoter and not of the MHRE mediate inhibitory effects on *PLAT* expression.

## Material and Methods

### Cells

HUVEC were isolated de novo as previously described [24,25] and cultured in EGM2 medium (Cambrex); peripheral blood monocytes were isolated from blood buffy coat by cold aggregation [26] and cultured in RPMI1640 and 10% fetal bovine serum (FBS); human foreskin fibroblasts were generated from skin biopsies and cultured in Dulbecco’s modified Eagle’s medium (DMEM) + 10% FBS, as previously described [27]; human astrocytes and hepatocytes were purchased from Lonza and cultured in AGM and HCM medium (Lonza), respectively. Bowes human melanoma cells, Huh7 and HepG2 human hepatoma cells and HT1080 fibrosarcoma cells were obtained from the DSMZ German Collection of Cell cultures and cultured in Dulbecco’s modified Eagle’s medium with 10% FCS; HeLa cervical cancer cells and NB4 acute promyelocytic leukemia cells were from the DSMZ and grown in RPMI, 10% FCS. The identity of all cell lines was authenticated by DNA profiling (performed at the DSMZ) and no rodent mitochondrial DNA sequences could be detected. None of the cells studied had sequence variants in the proximal t-PA gene promoter.

### Measurement of t-PA antigen concentrations

Concentrations of t-PA antigen were measured by ELISA, as previously described [28].

### t-PA mRNA analysis

Levels of t-PA mRNA in the different cell types were measured by the quantitative reverse transcriptase real time PCR (qPCR). Total cellular RNA was isolated using the Trizol reagent (Invitrogen) and reverse transcribed using Improm-II reverse transcriptase (Promega) according to the manufacturer’s instructions. Thereafter, qPCR was performed as described previously [29] using the ΔΔCT method. The expressed Alu repeat (EAR) sequence was used for normalization of qPCR results [30]. The oligonucleotide sequences used for qPCR are given in Table 1. Levels of the t-PA mRNA in the different cell types were compared to HUVEC taken as reference cells.

**Table 1:** Oligonucleotides used in this study

Experiment		Location^#^
EMSA^≠^	• CRE oligo 5′-TTCCTG**CG**ATTCAATGACATCA**CG**GCTGTG-3’ • Mutant CRE oligo 5’TTCCTG**CG**ATTCAGAATTAGTG**CG**GCTGTG 3’	-127 to -97
	• Sp1 oligo 5’GCCAC**CG**ACCCCACCCCCTGCCTGGA-3’ • Mutant Sp1 oligo 5’GCCAC**CG**TCTTGATCTCCTGCCTGGA3’	+53 to +79
DNA methylation analysis	Outer	
	• t-PA prom 10 F 5’TTTGAAAAGGTGTTAGTAAG3’ • t-PA prom 10 R 5’ACCACTAAAAAAACAAAACC3’	-721 to -382
	• t-PA prom12F2 5’GTTATTATAGGGTTTTGAAAG3’ • t-PA prom12R 5’AAAAAAACAAACCCCAAAATACAA3’	-217 to +220
	• t-PA prom 14 F 5’TTTGGGTTTATTTAAGGGGATGT3’ • t-PA prom14R 5’AAAAATTTTCTCTCCAACCCTAAAC3’	-577 to +156
	• t-PA enh 23 F 5’GAGAGAGGAGTTATGGAAAG3’ • t-PA enh 23 R 5’TACATATAATCCCAACTACT3’	-7538 to -6992
	• t-PA enh27F 5’ATTATTGTATTTTAGGTAGGGTG3’ • t-PA enh27R 5’CCTTCCTAAATCAAACATTTTT3’	-8118 to -7470
	Inner	
	• t-PA prom11F 5’TAAGGGAAATGGTTTGTTTA 3’ • t-PA prom 11 R 5’CTACRATAAAAAATACCCCCATA3’	-704 to -416
	• t-PA prom 13 F3 5’TTTTTAAGTTTGGGATATTAGGA3’ • t-PA prom13R 5’AAAATTTTCTCTCCAACCCTAAACT3’	-193 to +155
	• t-PA prom15 F5’GAGGTTATTTATTGTAGTTTTGTATTTTAT3’ • t-PA prom15 R 5’CAACTCTAAACTCCCCACAACTC 3’	-535 to +118
	• t-PA enh 23F 5’GAGAGAGGAGTTATGGAAAG3’ • t-PA enh 22R2 5’ATCCCAAACCATAACTATAT3’	-7538 to -7216
	• t-PA enh 21F 5’AGTTTTTGTTGTGGAAGTTA3’ • t-PA enh 21 R2 5’ATAAAAAAATCCCTTAAACC 3’	-7353 to -7024
	• t-PA enh28F 5’GTGTTTTTTTATTTGATTTATGTT3’ • t-PA enh28R 5’ ATAACTTTCCATAACTCCTCTCT3’	-8048 to -7515
RACE	• PLATgSp1R 5’CACACAGCAGCAGCACACAGCAGAGCCCTC 3’ • PLATgSp1innR 5’CAGAGCCCTCTCTTCATTGC3’ • Forward primers were provided with the 5’ RACE kit (Clontech) biotin-AGGGACGCTGTGAAGCAATCATGG	• -254 • -235 • -213
Reporter gene	t-PA prom 26 F 5’GTTTATGTGAGCAAACAGCAGA 3’	
Real time qPCR	• t-PA-F 5’ CCGGCTACGGCAAGCA 3’ • t-PA–R 5 ‘AGCGGCTGGATGGGTACA’3’ • EAR-F 5’-GAGGCTGAGGCAGGAGAATCG-3’ • EAR-R 5’-GTCGCCCAGGCTGGAGTG-3’	

^#^ The location indicates the position of the 5’ end of the oligo with respect to TSS1 of the t-PA gene or, for 5’RACE analysis, with respect to the mRNA sequence for a transcript starting at TSS1.

NB. F and R denote forward and reverse primers, respectively.

≠ For the electrophoretic mobility shift analysis (EMSA) only the forward oligo sequence is given. Underlined are the CRE or Sp1 core recognition sites. In bold the CpGs near the core sequences.

For RACE analysis the location is given with respect to TSS1. EAR = The expressed Alu repeat

### DNA methylation analysis

500 ng of DNA was modified with sodium bisulfite (MethylCode bisulfite conversion Kit, Invitrogen). The bisulfite-treated DNA was subjected to touchdown amplification from 65°C to 55°C with 30 cycles of PCR in a volume of 25 μl using the outer primers described in Table 1. After purification using the Illustra Exostar^TM^ kit (GE Healthcare), two μl of the PCR product were used as template for another nested PCR amplification using the inner primers. The secondary PCR products were purified and sequenced directly to determine their degree of cytosine methylation by comparing the ratio of cytosine and thymidine at the CpG positions, as previously described [19]. In one experiment the effect of 5-Azacytidine (Sigma-Aldrich) on DNA methylation of the proximal t-PA promoter was analyzed. To this end Huh7 cells were plated on six well dishes at 2.10^5^ cells per well and incubated for 3 days in presence or absence of 2μM 5-Azacytidine, Then DNA and total RNA were extracted to perform DNA methylation analysis and t-PA mRNA quantitation.

### Determination of the transcription start site by 5’ RACE analysis

5′ rapid amplification of cDNA ends (5’ RACE) analysis was employed to determine the transcription start site (TSS) of the *PLAT* gene for different cell types secreting appreciable amounts of t-PA. RNA was prepared (Trizol Reagent; Invitrogen) and 5′ RACE performed with the SMART^™^ RACE cDNA Amplification Kit according to the manufacturer’s instructions (Clontech). Briefly, 1 μg of RNA was used as starting material and 5′ RACE products amplified by PCR using the universal primer (UPM) included in the kit, and by a *PLAT* gene specific primer (Table 1, PLATgSp1R). For astrocytes and Bowes melanoma cells all amplification products were specific for t-PA. For HUVEC, HT1080 cells and HeLa cells it was necessary to purify the first PCR product by incubation with a biotinylated t-PA mRNA specific oligonucleotide followed by capture on streptavidin agarose and a second PCR using a nested primer (PLATgSp1innR). PCR products were purified by agarose gel electrophoresis and specific bands isolated using the PCR cleanup gel extraction kit (Macherey-Nagel). The 5′ ends were identified by direct DNA sequencing.

### Reporter gene plasmid constructs and in vitro methylation

DNA corresponding to the *PLAT* promoter region from -401 to +151 or to the region -151 to +151 was generated by direct DNA synthesis (Genecust) and inserted into the pCpGL plasmid, a CpG-free luciferase reporter vector [31]. Mutant plasmids in which selected CpGs in the region -151 to + 151 had been mutated were generated by direct DNA synthesis and inserted into the pCpGL plasmid (mutA: CpGs -121 and -106 mutated into AG and TG; mut B: CpGs -81, -51 and + 94 into TG, CC and CC; mutC: CpGs +27, +42, +50 and +59 into CC, CA, CA and CA). All insert DNA sequences were verified by DNA sequencing. The pCpGLprom401, pCpGLprom151 and the mutant plasmids were methylated by incubation with M.SssI (New England Biolabs) in the presence of 160 μM S-adenosylmethionine for 4 hours at 37°C. After the first two hours a further dose of 160 μM S-adenosylmethionine was added. The methylation status of the methylated plasmids, except mutC, was verified by restriction enzyme digestion using the enzyme HpaII, which does not cut the CpG of the recognition sequence CCGG (located at position +27) if it is methylated or using the enzyme MspI, which cuts both methylated and unmethylated CpG at this position. The restriction pattern was analyzed by agarose gel electrophoresis.

### Luciferase reporter gene assays

HT1080 cells or HeLa cells were plated in 96 well plates at 8000 cells per well and co-transfected, using the Lipofectamine LTX and Plus^TM^ reagent (Life technologies), with 80 ng of the various pCpGL plasmids and 20 ng of Renilla Luciferase Control Reporter plasmid (Promega) per well. The pCpGL plasmid containing no *PLAT* promoter sequence served as a negative control. In some experiments the protein kinase C activator phorbol 12-myristate 13-acetate (PMA)(Sigma-Aldrich) at a final concentration of 20 nM was added 24 hours after transfection. 36 hours after transfection, luciferase activity was measured using the Dual-Glo® Luciferase Reporter Assay System (Promega). Firefly luciferase activity of each individual transfection was normalized against Renilla luciferase activity. Each transfection was done in quadruplicate. Luminescence was measured using a Perkin Elmer Victor3 luminescence detection instrument (Perkin Elmer, Waltham, MA, USA).

### Electrophoretic Mobility Gel Shift Assay (EMSA)

To determine binding between transcription factors and DNA, an EMSA was performed. Nuclear extracts were prepared with the NE-PER nuclear extraction reagent (Pierce) and 5 μg of these incubated with 50 nM of IRD700 labeled double-stranded CRE or Sp1 oligonucleotides (Metabion International) (Table 1). Competition experiments were done using different concentrations of the corresponding double-stranded unmethylated or methylated oligonucleotides or double-stranded oligonucleotides in which the CRE site or the Sp1 sites have been mutated. After 20 minutes incubation at room temperature, complex formation of the IRD700 labeled oligonucleotides with proteins in the nuclear extract were analyzed in a 5% non-reduced and non-denaturing polyacrylamide gel according the indications of the manufacturer (Odyssey Infrared EMSA kit, LI-COR). The DNA-protein complexes were detected with an Odyssey Infrared scanner (Li-COR) and quantified using the Odyssey Infrared Imaging System (Software version 3.16).

For supershift assays, nuclear extracts were incubated with 1 μg of antibodies directed against specific transcription factors (polyclonal antibodies to c-fos, fra2 or junD or monoclonal antibodies to Sp1 or Sp3; all from Santa Cruz Biotechnology) at room temperature for 1 hour followed by incubation with IRD700 labeled oligonucleotides and competitor oligonucleotides for 20 minutes.

### Statistical analysis

Statistical analysis was performed using by one way analysis of variance (ANOVA) followed by the student’s t-test. A p value < 0.05 was considered statistically significant.

## Results

### Determination of the *PLAT* gene transcriptional start site in different human cell types

**Fig 1:**
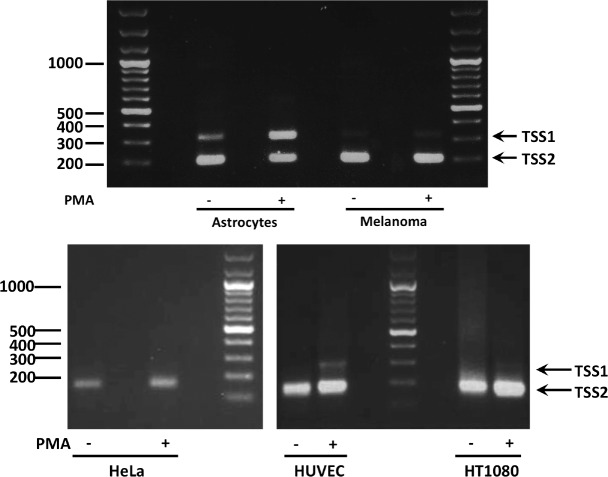
Determination of the *PLAT* gene transcriptional start site (TSS) in different human cell types. The TSS for the *PLAT* gene was assessed by 5’RACE analysis in different t-PA producing human cell types: astrocytes, Bowes melanoma cells, HeLa cells, HUVEC and HT1080 cells, both in cells incubated in culture medium alone (—) or in medium containing 20 nM PMA (+). RACE PCR products were analyzed by agarose gel electrophoresis. PCR products were sequenced and two distinct TSS’s were detected: TSS1 corresponding to position 42’207’676 on chromosome 8 in the GRCh38/hg38 assembly (http://genome.ucsc.edu/) and TSS2, located 110 bp downstream. Note that the intensity of the band corresponding to TSS1 was increased by PMA (20 nM for 24h) in astrocytes and HUVEC. This figure shows representative results for each cell type.

Before studying the influence of *PLAT* gene promoter/enhancer methylation on *PLAT* gene expression, we investigated the *PLAT* transcriptional start site (TSS) by 5’RACE analysis using total RNA from five t-PA expressing cell types: astrocytes, Bowes melanoma cells, HeLa cells, HUVEC and HT1080 cells (Fig 1). Two TSS’s have been described for the *PLAT* gene: TSS1 corresponds to position 42’207’676 on chromosome 8 in the GRCh38/hg38 assembly (http://genome.ucsc.edu/), whereas TSS2 is located 110 bp downstream [14,32,33]. Analyses were made on mRNA isolated both from non-stimulated cells and cells treated with PMA (20 nM) for 24h. Treatment with PMA increased t-PA antigen release by astrocytes, Bowes melanoma cells, HUVEC and HeLa cells, but decreased t-PA twofold in HT1080 cells (not shown). The latter is in agreement with previous observations by Costa et al. [34].

The 5’RACE analysis showed that astrocytes use both TSS1 (band at 300 bp) and TSS2 (band at 200 bp) and that PMA treatment increases the use of TSS1; in contrast Bowes melanoma cells only use TSS2 even after treatment with PMA (Fig 1). HUVEC, HT1080 and HeLa cells predominantly use TSS2. In HUVEC a minor band corresponding to TSS1 was present only after PMA treatment (Fig 1). For the remainder of this article we designate nucleotide positions with respect to TSS1, which is the position given in the University of California Santa Cruz Genome Browser, Assembly GRCh37/hg19 (http://genome.ucsc.edu/).

### Methylation of the *PLAT* promoter and the multihormone responsive enhancer (MHRE) and their relationship with t-PA secretion and t-PA mRNA levels

**Fig 2:**
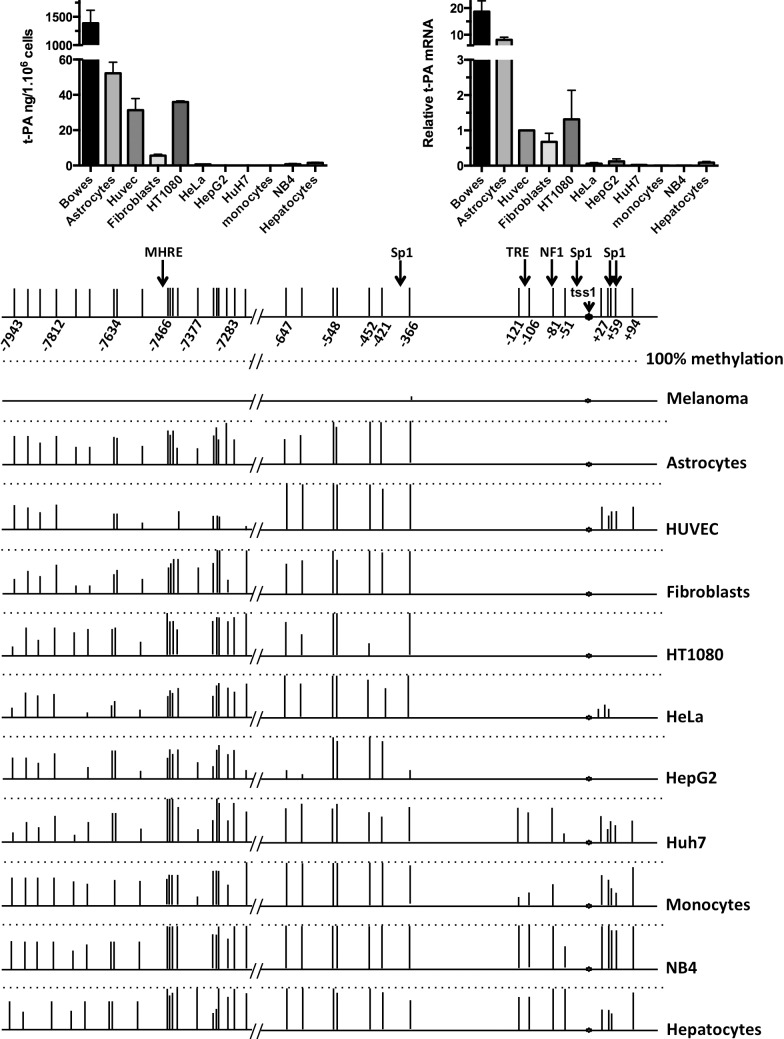
Secretion of t-PA, t-PA mRNA and methylation of the *PLAT* promoter and the multihormone responsive enhancer (MHRE) in different cell types. Secretion of t-PA and t-PA mRNA, as well as the methylation state of the promoter region and the MHRE was analyzed for five different human primary cell types (endothelial cells, astrocytes, fibroblasts, monocytes and hepatocytes). These primary cells were used in single anonymous donor experiments. Results were averaged from at least three donors for HUVEC, monocytes and fibroblasts and two donors for astrocytes and hepatocytes). Furthermore, six different human transformed cell lines were used (Bowes melanoma cells, HT1080 fibrosarcoma cells, HeLa cells, HepG2 cells and Huh7 hepatoma cells, and NB4 acute promyelocytic leukemia cells).

We set out to analyze if a relationship could be found between t-PA expression measured in cell-conditioned medium and at the mRNA level and the methylation state of 16 CpG residues in the proximal *PLAT* gene promoter (from -647 to + 94 with respect to the TSS1) and 19 CpG residues near the MHRE (from – 7943 to -7227). Antigen levels of t-PA were determined in 24h-conditioned medium of five human primary cell types (endothelial cells, astrocytes, fibroblasts, monocytes and hepatocytes) and six human transformed cell lines (Bowes melanoma cells; HT1080 fibrosarcoma cells, HeLa cells, HepG2 and Huh7 hepatoma cells, and NB4 acute promyelocytic leukemia cells). By far the highest degree of t-PA secretion was observed for Bowes melanoma cells. Intermediate t-PA secretion was obtained for astrocytes, HUVEC, HT1080 cells and fibroblasts, and low or absent t-PA secretion from the other cell types (Fig 2 top left). tPA mRNA levels measured by qPCR showed the highest expression in Bowes melanoma. The results obtained for the different cell types reflect the t-PA antigen levels (Fig 2 top right). In DNA from Bowes melanoma cells we detected no CpG methylation in the promoter region from -647 to +94. For the other cell types two distinct zones could be identified: the upstream promoter region from -647 to -366, in which all CpGs were methylated by more than 80% in all cell types analyzed (except HepG2 cells, which had 3 out of 7 CpGs mostly unmethylated), and the proximal promoter region -121 to +94 which showed a more diverse pattern of CpG methylation. Three different patterns were observed: intermediate t-PA secretion and most of the CpGs unmethylated (astrocytes, HUVEC, HT1080 cells and fibroblasts), low t-PA secretion and most of the CpGs unmethylated (HeLa cells and HepG2 cells) and low t-PA secretion and most of the CpGs methylated (Huh7 cells, monocytes, NB4 cells and hepatocytes) (Fig 2 bottom). These results suggest that an unmethylated state of the promoter between -121 to +94 is required, but not sufficient for intermediate t-PA secretion.

Top left: Secretion of t-PA, expressed in ng per million cells per 24h in cells cultured in medium alone (mean ± SEM of at least four independent measurements). Top right: t-PA mRNA levels in the different cell types, expressed as relative to t-PA mRNA levels in HUVEC, using EAR mRNA for normalization of the results. Bottom: This part of the figure gives the percentage methylation of the CpGs in the *PLAT* promoter region (between -647 and + 94, with respect to TSS1) and the MHRE (between -7943 and -7283) in the different cell types. Bar length’s are proportional to the percentage methylation for each position, with the dotted lines indicating 100% methylation. The position of TSS1, of the promoter interaction sites with CRE, different Sp1’s and NF1, as well as the position of the MHRE is indicated at the top of the figure. Note the extremely high secretion rate of t-PA from and t-PA mRNA levels in Bowes melanoma cells and the complete absence of CpG methylation in the *PLAT* promoter region and near the MHRE with these cells.

We also analyzed the methylation state in the region (from – 7943 to -7227) near the multihormone responsive enhancer (MHRE). Again no CpG methylation was observed for the Bowes melanoma cells in this region, whereas for the other cell types no correlation could be determined between t-PA secretion and the degree of CpG methylation (Fig 2 bottom).

**Fig 3:**
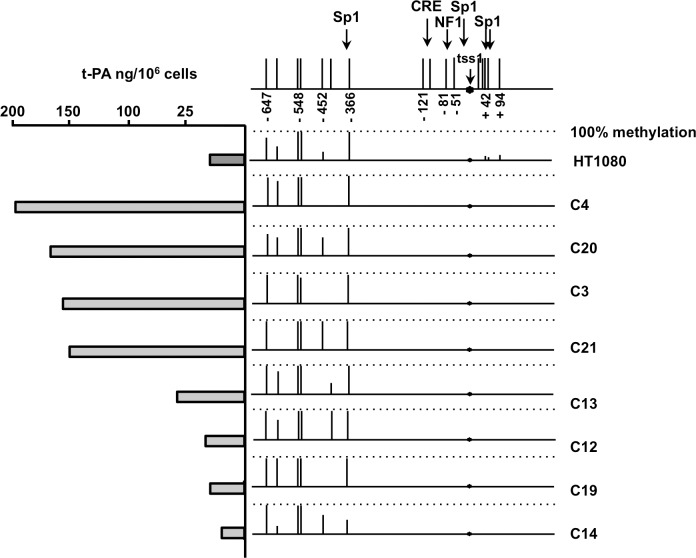
Methylation of the *PLAT* promoter and t-PA secretion in HT1080 cell clones. The methylation state of the promoter region was analyzed for eight HT1080 cell clones differing in their t-PA secretion and in non-cloned HT1080 cells. The figure gives the percentage methylation of the CpGs in the *PLAT* promoter region (between -647 and + 94, with respect to TSS1). Bar length’s are proportional to the percentage methylation for each position, with the dotted lines indicating 100% methylation. The position of TSS1 and of the promoter interaction sites with CRE, Sp1 and NF1 is indicated at the top of the figure. Secretion of t-PA expressed in ng per million cells over a 24h period by cells cultured in medium alone is given at the left of the figure.

To further investigate the relationship between *PLAT* proximal promoter methylation and t-PA secretion, we compared these parameters in clones derived from HT1080 cells. Indeed, we have previously shown that HT1080 cells show a large clonal variation with respect to t-PA secretion [35]. Fifteen stable HT1080 clones were obtained by limiting dilution. Expression of t-PA was analyzed by measuring t-PA antigen in 24h-conditioned medium from each clone. DNA methylation in the region -647 to +94 was measured for the four highest t-PA secreting clones and the four lowest t-PA secreting clones. The pattern of CpG methylation was comparable for all clones and similar to that of the parent HT1080 cell line (Fig 3).

### Effect of 5-Azacytidine treatment on t-PA mRNA in Huh7 cells

**Fig 4:**
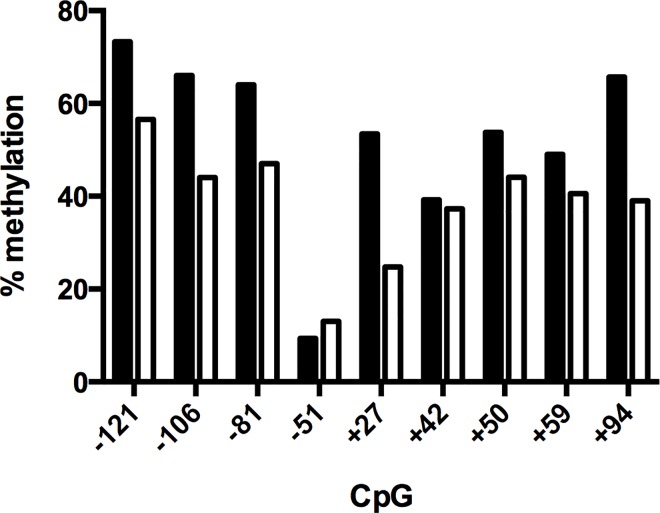
Effect of 5-azacytidine treatment on the degree of methylation of CpG residues in the proximal t-PA promoter profile in the proximal promoter. Huh7 were cultured for 3 days in the absence (black bars) or presence (white bars) of 2 μM azacytidine. Bar lengths are proportional to the percentage methylation for each position.

Treatment of Huh7 with 5-azacytidine results in a 2.3 +/- 0.4 fold increase in t-PA mRNA (mean ± SE, n = 3). Methylation analysis performed in parallel in the same sample showed that azacytidine reduced the degree of methylation of the various CpG residues in the proximal promoter (-121 to +94) (Fig 4).

### Effects of DNA methylation on *PLAT* proximal promoter-driven reporter gene activity

**Fig 5:**
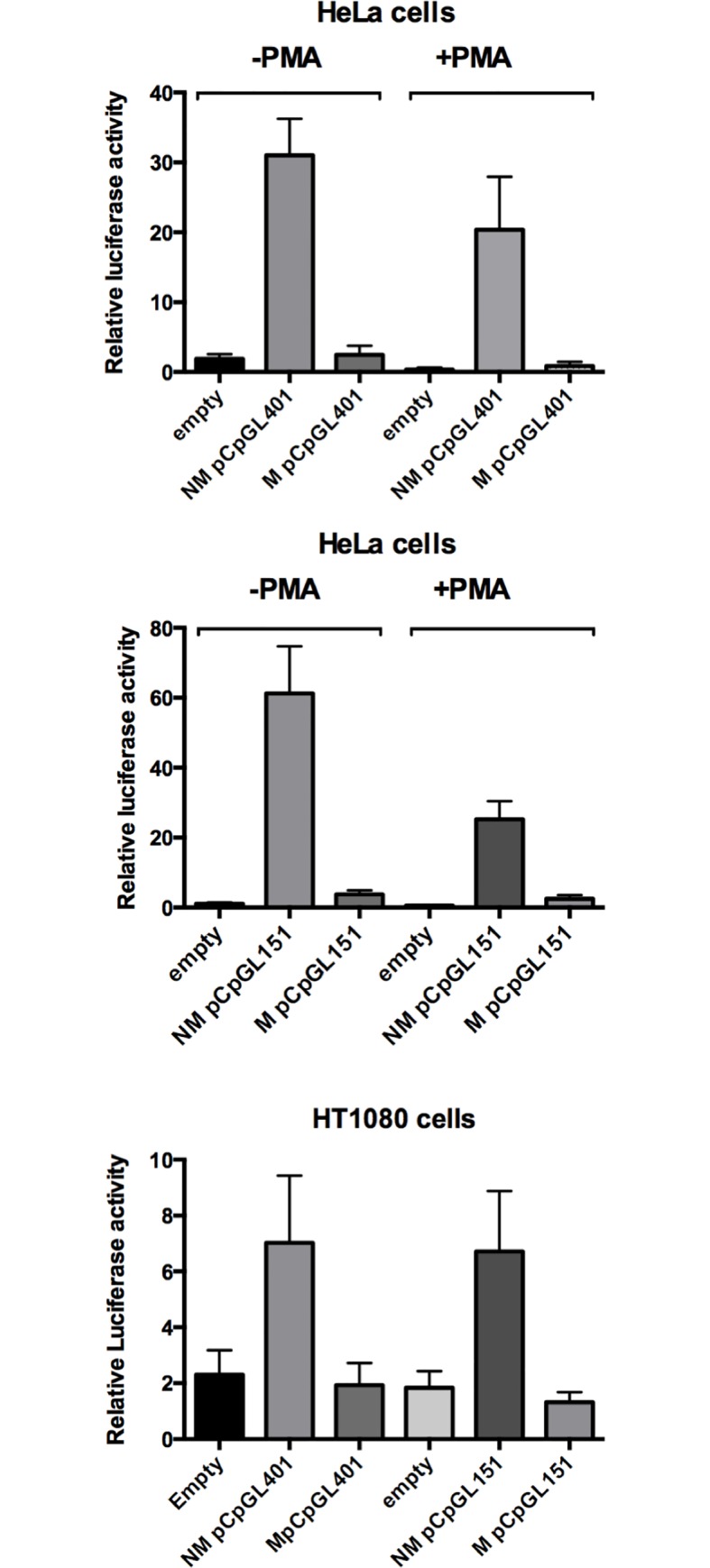
Effect of DNA methylation on *PLAT* promoter-driven reporter gene activity. Reporter gene activity of methylated (M) or unmethylated (NM) plasmids was compared after transfection of HeLa cells (top and center) or HT 1080 (bottom) with pCpGLprom401 and pCpGLprom151 plasmids, which contain the *PLAT* promoter region from -401 to +151 and -151 to +151, respectively, linked to a CpG-less firefly luciferase gene. Note that reporter gene activity of the methylated plasmids was the same as that of reporter gene plasmids lacking a promoter (empty) and markedly reduced compared to unmethylated plasmids. Results are presented as means ± SEM of 3 to 4 independent experiments.

We investigated the effect of CpG methylation on *PLAT* gene activity in the context of *PLAT* proximal promoter driving reporter gene expression. For this study we used a firefly luciferase reporter plasmid pCpGL [31], which is devoid of CpG residues. Reporter gene activities of unmethylated pCpGL-PLATprom401 and pCpGL-PLATprom151 plasmids (containing the promoter region from -401 to +151 and from -151 to + 151, respectively) transfected into control HeLa cells or HT1080 cells were comparable (Fig 5). Methylation reduced reporter gene activity of both plasmids to levels comparable to that of an insert-less pCpGL plasmid (Fig 5).

### Influence of DNA methylation on transcription factor binding to the proximal *PLAT* promoter

Previous research [12,13] has shown that two sites in the proximal promoter are of particular importance for *PLAT* gene expression, a CRE site located at—113 to – 106, and an Sp1 binding site (also named GC box) at +62 to +69. The CRE is closely flanked by two CpG sites and the Sp1 site is directly preceded by one CpG site. To study transcription factor binding to these sites, we performed electrophoretic mobility shift analysis (EMSA) using nuclear extracts from HUVEC, Bowes melanoma cells, HeLa cells and HT1080 cells, with or without PMA stimulation (20 nM, 24h).

**Fig 6:**
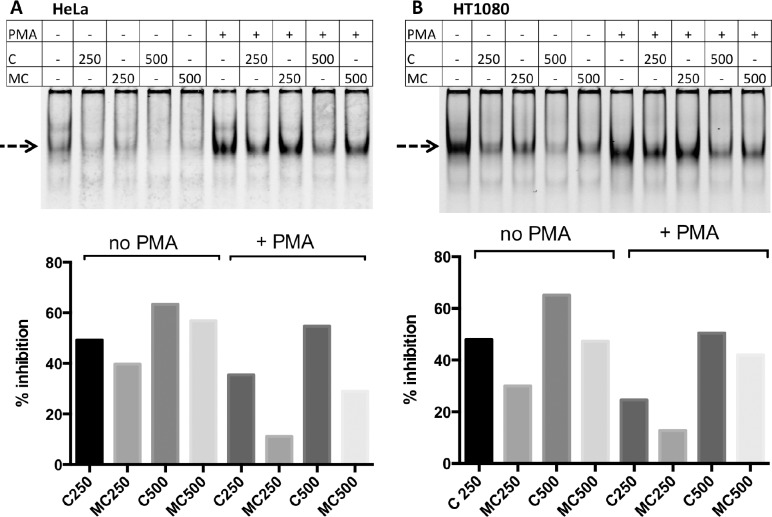
Influence of DNA methylation on transcription factor binding to a double-stranded DNA containing the CRE site. The effect of oligonucleotide methylation on transcription factor binding was assessed using nuclear extracts from HeLa or HT1080 cells and by comparing the effect of different concentrations of unmethylated (C, concentrations given in nM) or methylated competitor oligo-nucleotides (MC) on the binding of transcription factors to the double-stranded IRD700-labeled unmethylated oligonucleotide TTCCTG**CG**ATTCAATGACATCA**CG**GCTGTG, which includes the CRE site (underlined) flanked by two CpGs (in bold). Note that the methylated oligonucleotide was capable of reducing band intensities, albeit at a slightly lesser efficacy as the unmethylated oligonucleotide. The dashed arrow indicates the band whose intensity increases with PMA treatment of HeLa cells. The top of the figure shows the EMSA images and the bottom the % inhibition of band intensities when nuclear extracts were co-incubated with the indicated concentrations (in nM) of unmethylated (C) or methylated (MC) unlabeled competitor oligonucleotides.

Incubation of nuclear extracts from the different cell types with annealed IRD700 labeled double-stranded oligonucleotide containing the *PLAT*-CRE produced several shifted bands. Almost no shifted bands were observed in the presence of an excess (10^−5^ M) of unlabeled double-stranded CRE competitor oligonucleotide (UC) (S1 Fig). An excess of unlabeled oligonucleotide in which the CRE was mutated did not reduce binding of the labeled CRE oligonucleotide (not shown). Treatment of Bowes melanoma cells and HeLa cells with PMA markedly increased the intensity of one of these bands, whereas for HUVEC and HT1080 cells, PMA treatment did not change the pattern of protein binding (S2 Fig and Fig 6, marked with a dashed arrow).

An antibody shift experiment was performed by adding antibodies to c-fos, fra2 or junD to the mixture of nuclear extracts and the *PLAT*-CRE probe. Incubation with antibodies against JunD, but not with antibodies against c-fos or fra2, resulted in the formation of an additional slower migrating band (S2A to S2D Fig, solid arrow). A minor additional slower migrating band was observed for nuclear extracts of HeLa and HT1080 cells (S2C and S2D Fig, dotted arrow). Incubation with anti-junD antibodies did not result in a clear depletion of one of the EMSA bands. This implies that other, thus far unidentified, transcription factors bind to the CRE probe. No difference was observed in the intensity of junD supershift band formation between non-treated and PMA-treated cells. This result indicates that PMA modifies the pattern of binding of one or several transcription factors other than JunD.

We investigated to what extent methylation of the two CpGs in the double-stranded CRE oligonucleotide affected transcription factor binding to the CRE probe. For this we used nuclear extracts from HeLa and HT1080 cells, both under basal conditions and after stimulation with PMA and performed EMSA using the IRD700-labeled CRE probe and different concentrations of methylated or unmethylated unlabeled CRE probe. Quantification of the intensity of the main shifted band (dashed arrow, Fig 6 top) showed that the methylated CRE competitor (MC) was capable of reducing transcription factor binding to the IRD700 labeled CRE probe, although its efficacy appeared to be less than that of the non-methylated CRE probe (C) (Fig 6 bottom). Note that at the concentrations of methylated or unmethylated competitor oligonucleotide used the intensity of the PMA-induced band (arrow) was reduced in HeLa cells or HT1080 cells but not fully abolished.

**Fig 7:**
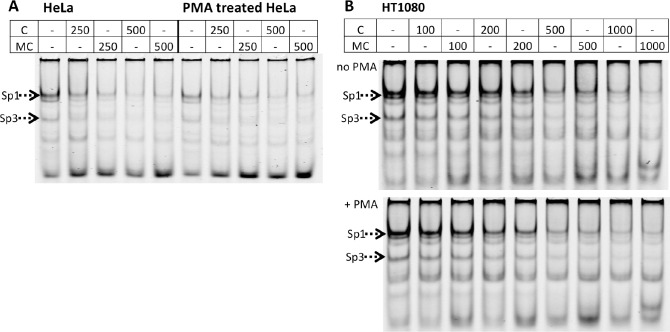
Influence of DNA methylation on transcription factor binding to a double-stranded DNA containing the Sp1 site. The effect of oligonucleotide methylation on transcription factor binding was assessed using nuclear extracts from HeLa or HT1080 cells and by comparing the effect of different concentrations of unmethylated (C, concentrations given in nM) or methylated competitor oligonucleotides (MC) on the binding of transcription factors to a double-stranded IRD700-labeled unmethylated oligonucleotide GCCAC**CG**ACCCCACCCCCTGCCTGGA, which includes the Sp1 site at +62 (underlined) immediately preceded by one CpG (in bold). The analysis was done for cells cultured for 24h in culture medium in the absence or presence of 20 nM PMA. The effect of oligonucleotide methylation on transcription factor binding was assessed using nuclear extracts from HeLa cells (A) or HT1080 cells (B) and by comparing the effect of different concentrations of unmethylated (C, concentrations given in nM) or methylated competitor oligonucleotide (MC) on band intensities. Note that the methylated and unmethylated oligonucleotides had comparable efficacies in preventing transcription factor binding to the IRD700 labeled unmethylated Sp1 probe.

Incubation of nuclear extracts of HT1080 cells, HeLa cells, HUVEC or Bowes melanoma cells with a double-stranded IRD700 labeled probe for the GC-rich sequence at +62 to +69, which contains an Sp1-binding site (henceforth named Sp1 probe) produced at least five shifted bands. The upper four bands were strongly reduced in the presence of an excess (10^−5^ M) of unlabeled double-stranded Sp1 probe (S1 Fig), while mutant probes had no effect (not shown). Co-incubation of nuclear extracts and the Sp1 probe with antibodies against Sp1 resulted in the formation of a clear supershifted band with nuclear extracts from HT1080 cells, HUVEC and Bowes melanoma cells but not from HeLa cells. The intensity of the band seen without Sp1 antibodies decreased when Sp1 antibodies were used (dotted arrow in S3A and S3B Fig). No difference was seen between non-treated and PMA-treated cells. The same experiments using anti-Sp3 antibodies resulted in a clear supershift with the nuclear extracts from all four cell types and again the band seen without antibodies decreased (dotted arrow in S3C and S3D Fig). The inhibition of transcription factor binding by 500 or 1000 nM of unmethylated or methylated Sp1 probes was comparable for the two cell types tested, HeLa and HT1080, both for PMA treated or non-treated cells (Fig 7A and 7B).

### Effect of DNA methylation on reporter gene activity from mutated *PLAT* promoters

**Fig 8:**
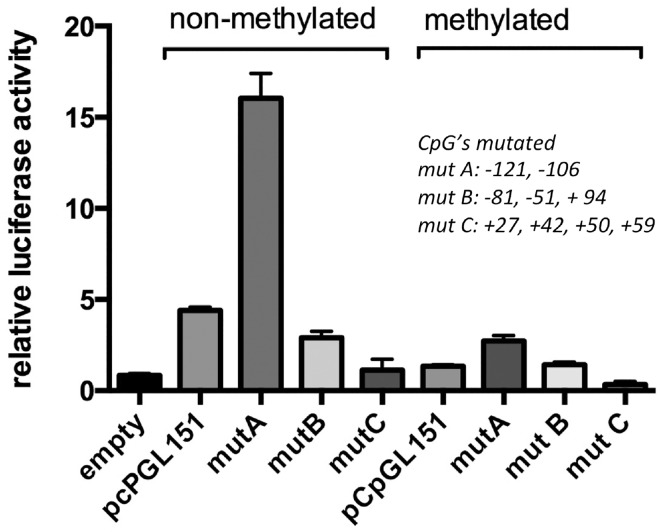
Effect of DNA methylation on reporter gene activity from mutated *PLAT* promoters. Reporter gene activity was compared after transfection of HT1080 cells with pCpGLprom151 and promoter mutants. MutA: CpGs -121 and -106 mutated; mut B: CpGs -81, -51 and + 94 mutated; mutC: CpGs +27, +42, +50 and +59 mutated. Results are expressed as means ± SEM of four independent experiments.

To determine whether removal of specific CpG residues resulted in abolition of the inhibitory effect of CpG methylation, we compared reporter gene activity of the pCpGL-PLATprom151 plasmid with that of three pCpGL-PLATprom151 plasmids having specific CpGs mutated. Mutation of the CpG residues at -121 and -106 flanking the CRE (mutA) resulted in a threefold increased reporter gene activity. Methylation of mutA reduced reporter gene activity by over 80% (Fig 8). Mutation of the CpG residues at -81, -51 and + 94 (mutB) had no effect on reporter gene activity of the non-methylated construct and methylation reduced its activity by 80% (a level comparable to that of methylated wild type pCpGL-PLATprom151) (Fig 8). Mutation of the four CpGs located between +27 and +59 (mutC), markedly reduced reporter gene activity of the non-methylated plasmid and methylation further reduced its reporter gene activity (Fig 8).

### Effect of trichostatin on nonmethylated and methylated *PLAT* promoter-driven reporter gene activity

**Fig 9:**
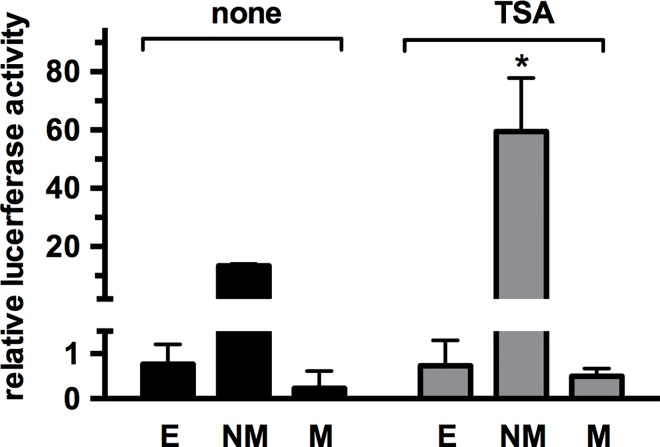
Effect of trichostatin on nonmethylated and methylated *PLAT* promoter-driven reporter gene activity. HT1080 cells were transfected with nonmethylated (NM) and methylated (M) pCpGLprom151 plasmids and reporter gene activity measured in the absence or presence of 3 μM trichostatin (TSA). The empty condition represents reporter gene activity of a pCpGL plasmid lacking a promoter insert. Note that reporter gene activity of methylated pCpGL plasmids was not significantly different from that of the empty plasmids. Results are expressed as means ± SEM of four independent experiments. * p< 0.05

Methylation of CpG islands may attract co-repressor complexes that contain histone deacetylases [36,37]. To determine whether this mechanism contributes to the inhibitory effect of methylation on the activity of the *PLAT* promoter, which is not part of a CpG island, we transfected HT1080 cells with unmethylated or methylated pCpGL-PLATprom151 plasmid and studied the effect of the HDAC inhibitor trichostatin. Treatment with trichostatin, induced a fourfold increase in reporter gene activity of the unmethylated pCpGL-PLATprom151 plasmid, but did not increase reporter gene activity of the methylated pCpGL-PLATprom151 plasmid, which remained comparable to that of the insert-less pCpGL plasmid (Fig 9).

## Discussion

We investigated the mechanisms by which DNA methylation in the proximal promoter and the multihormone responsive enhancer affects *PLAT* gene expression in different human primary cells and transformed cell lines. To be able to draw firm conclusions from promoter methylation studies it is essential to first define the position of the transcriptional start site (TSS) used by a particular cell type. Indeed, alternative TSS’s have been described for a large number of human protein-coding genes, which may reflect alternative promoter usage [38,39]. Two TSS’s located 110 bp from each other have been described for the *PLAT* gene [14,32,33]: TSS1 has been described for PMA treated HeLa cells [28], and TSS2, which is located 110 base pairs downstream of TSS1, in human WI-38 human fetal fibroblasts [33] and a cell line derived from human endothelial cells [14]. In a subset of the primary cells and cell lines used, that are capable of releasing high or intermediate quantities of t-PA (Fig 2), we determined which of these two TSS’s, or others, is used. We observed that in untreated astrocytes the shorter transcript (TSS2) was the principal transcript, with a small contribution of TSS1, whereas after PMA stimulation both TSS1 and TSS2 were used to the same extent. In contrast, in Bowes melanoma cells, HT1080 cells and HeLa cells, TSS2 was exclusively used even after PMA treatment. For HUVEC we observed a small contribution of TSS1, but only after PMA treatment. Our results with PMA-stimulated HeLa cells are in contrast to previous results [32,40], which demonstrated the preferential use of TSS1. At the present time it is not possible to establish whether these differences are due to the use of a different variant of HeLa cells or to different cell culture conditions. As both TSS1 and TSS2 are located within the untranslated first exon of the *PLAT* gene, these alternative transcriptional start sites would not lead to differences in the *PLAT* translation product. It remains to be established by what mechanisms PMA treatment leads to an increased use of TSS1 and whether the alternative TSS use modifies *PLAT* gene responses.

The methylation state of the *PLAT* gene was investigated for two DNA regions: the proximal promoter region stretching from position -647 to +94 with respect to TSS1 and a stretch of 19 CpGs near the MHRE, which mediates *PLAT* gene transcriptional responses to steroid hormones and retinoic acid [17]. Also, the latter region contains a polymorphism that is associated with differences in the secretion rate of t-PA from the forearm [41]. A striking finding was the sharp distinction between the region between -647 and -366, which is almost fully methylated in all cell types, except Bowes melanoma cells, and the region -121 to +94, which is mostly unmethylated in all cell types secreting t-PA to a significant extent. This difference in methylation between the upstream promoter and the proximal promoter was previously shown in HUVEC [19,23] is observed for all cell types producing intermediate t-PA secretion. By comparing t-PA secretion from different cell types we observed that t-PA secretion by the Bowes melanoma cells was 50-fold higher than that of the second highest t-PA producer cell type. It remains to be established whether the upstream promoter region between -647 and -366 contains a methylation dependent suppressor element. The extremely high levels of t-PA secretion from Bowes melanoma cells was crucial for the development of t-PA as a thrombolytic agent for myocardial infarction and stroke [42]. The results obtained with the other cells type show that a mostly unmethylated proximal promoter (region from -121 to +94 with respect to TSS1) is required, but not sufficient for intermediate level t-PA secretion. It seems likely that the cell types with an unmethylated promoter and releasing low levels of t-PA lack other factors that are required for efficient t-PA secretion. In agreement with well-established findings for a large number of other genes [43–46], a methylated *PLAT* promoter is associated with low or absent t-PA secretion. Our results, however, imply that the effect of DNA methylation concerns only a very small region of the promoter stretching from -121 to +94. In previous studies we observed a large variation of t-PA secretion by HT1080 cell-derived clones [35]. In the present study we established that the proximal promoter (-121 to +94) was unmethylated in both the high t-PA producing HT1080-derived clones and in the low producer clones and that the CpGs in the region from -647 to -366 were all fully methylated. Our results thus show that differences in t-PA secretion by these clones is not due to differences in DNA methylation in the proximal promoter region.

In the present study we did not observe a relation between the methylation state of the MHRE and t-PA gene expression by the different cell types. However, MHRE methylation is likely to be important for t-PA gene expression since Magnusson et al [23] observed that placing HUVEC in cell culture leads to a reduction in MHRE methylation and an increase in t-PA expression.

Potentially the effect of CpG methylation on transcription factor binding may depend on the organization of the promoter region or may negatively affect the assembly of the RNA polymerase complex. To investigate this aspect we studied the effect of DNA methylation on luciferase reporter gene activity, using the pCpGL luciferase reporter plasmid, which is devoid of CpG residues. This enabled us to compare reporter gene plasmids that only differ by the methylation of CpG residues in the *PLAT* promoter. Reporter gene activity of plasmid pCpGL-PLATprom401, which contains DNA corresponding to the region -401 to +151 and pCpGL-PLATprom151 (region -151 to +151) exhibited similar reporter gene activity when transfected into HT1080 cells and methylation of both resulted in a similar reduction of reporter gene activity. From this we conclude that the region from -151 to +151, which possesses the CRE and the Sp1 binding sites, contains the principal elements required for promoter activity and the region that conveys sensitivity to the inhibitory effect of CpG methylation.

Our results suggest that the methylation state of the region -121 to +94 has a profound effect on *PLAT* expression. We hypothesized that DNA methylation of specific CpGs in this region may affect transcription factor binding to regulatory elements. We therefore investigated by EMSA to what extent DNA methylation influences transcription factor binding. We concentrated our efforts to two well-established regulatory elements in the proximal *PLAT* promoter: the CRE at -113 to -106 and the Sp1 site at +62 [12–15]. By using a comparative supershift analysis with nuclear extracts from Bowes melanoma cells, HUVEC, HeLa cells and HT1080 cells, with or without PMA treatment, we identified binding of junD to the CRE and binding of both Sp1 and Sp3 to the Sp1 site. PMA stimulation increased the intensity of one complex band after incubation of nuclear extracts of melanoma or HeLa cells with the CRE probe but did not modify the pattern of protein binding to the Sp1 site. Methylation of the two CpGs in the competitor double-stranded oligonucleotide for the CRE site modified its ability to compete for protein binding to the CRE site. However, the difference in efficacy was at most twofold, which appears insufficient to explain the complete lack of t-PA expression in cells with a methylated proximal *PLAT* promoter. In contrast, a previous study observed that methylation of a CpG located in the middle of a double-stranded oligonucleotide containing a consensus CRE site completely interfered with its ability to compete for transcription factor binding to a labeled CRE oligonucleotide and resulted in loss of transcriptional activity in vivo [47]. The results obtained for the Sp1 site are in accordance with a previous study that showed that binding of Sp1 was unaffected by methylation of an artificial Sp1 binding site [48].

To determine whether a particular CpG residue has a disproportionate contribution to this inhibitory effect we compared reporter gene activity of pCpGL-PLATprom151 with that of three reporter gene plasmids, one mutated at the CpGs surrounding the CRE (position -121 and -106), one mutated at the CpGs associated with the Sp1 binding site (residues +27 and +59) and with mutated at CpG -81, located in the NF1/CTF binding site for which repressor activity had been reported [13,49], as well as the CpGs -51 or +96. We observed that removal of the two CpGs near the CRE resulted in a fourfold increase in reporter gene activity. This suggests that one (or both) of these CpGs has a methylation independent, inhibitory effect on CRE-dependent gene transcription. Whether these CpGs interact with the NF1/CTF repressor element binding located 20 bp downstream of the CRE remains to be established. In contrast, mutation of the four CpGs between +27 and +59 near the Sp1 site resulted in a fourfold decrease in reporter gene activity. This suggests that one or more of the CpGs in this region are required for Sp1/Sp3-dependent transcription. As promoter methylation reduced reporter gene activity of all the mutant plasmids by more than 80%, we may conclude that the inhibitory effect of DNA methylation is not associated with a particular CpG residue in the proximal *PLAT* promoter.

Attraction of HDAC containing co-repressors to promoters containing methylated CpG islands has been reported as a mechanism of CpG methylation-dependent down regulation of gene activity [36,37]. We observed that treatment with the HDAC inhibitor trichostatin increased reporter gene activity more than fourfold. This is comparable to previous findings on the effect of HDAC inhibition on t-PA mRNA levels in endothelial cells [19,20,28,50]. However, HDAC inhibition did not restore reporter gene activity of methylated reporter genes. Therefore, the effect of DNA methylation on reporter gene activity from the proximal *PLAT* promoter is not principally due to the attraction of HDACs.

In conclusion, our results show that efficient *PLAT* gene expression requires a short stretch of unmethylated CpG’s in the proximal promoter. The relevance of proximal PLAT promoter methylation in various physiological and pathological conditions needs to be established in specifically designed studies that address each condition. This will require PLAT gene methylation analysis in the different cell types that contribute to local t-PA concentrations in physiological and pathological conditions such as diabetes [51,52] or neurodegenerative diseases [53,54], while taking into account environmental factors such as diet [55], exercise [56] or other relevant factors.

## Supporting Information

S1 Fig Effect of unlabeled double stranded oligonucleotides on transcription factor binding to the CRE-site or the Sp1-site. Top: Effect of an excess (10^−5^ M) of unlabeled double-stranded CRE oligonucleotide on the binding of transcription factors to the double-stranded IRD700-labeled unmethylated oligonucleotide TTCCTG**CG**ATTCAATGACATCA**CG**GCTGTG, which includes the CRE site (underlined) flanked by two CpGs (in bold). Note the reduction in intensity of the principal shifted bands. Bottom: Effect of an excess (10–5 M) of unlabeled double-stranded oligonucleotide containing the Sp1 site on the binding of transcription factors to the double-stranded IRD700-labeled unmethylated oligonucleotide GCCAC**CG**ACCCCACCCCCTGCCTGGA, which includes the Sp1 site at +62 (underlined) immediately preceded by one CpG (in bold). Note the reduction in intensity of the four upper bands.
(TIFF)

S2 Fig Identification of transcription factor binding to double-stranded DNA oligonucleotides containing the CRE site. A-D) A supershift experiment was performed to assess binding of c-fos, fra2 and junD in nuclear extracts from HUVEC (A), Bowes melanoma cells (B), HeLa cells (C) and HT1080 cells (D) to the doublestranded IRD700-labeled unmethylated oligonucleotide TCCTG**CG**ATTCAATGACATCA**CG**GCTGTG, which includes the CRE site (underlined) flanked by two CpGs (in bold). The analysis was done for cells cultured for 24h in culture medium in the absence (-) or presence (+) of 20 nM PMA. Note that in the presence of a large excess (10–5 M) of unlabeled competitor oligonucleotide (UC) almost no shifted bands were observed. The solid arrow and the dotted arrow indicate the supershift bands obtained with antibodies to junD. The dashed arrow shows the position of a band whose intensity increases with PMA treatment of Bowes melanoma cells or HeLa cells.
(TIFF)

S3 Fig Identification of transcription factor binding to double-stranded DNA oligonucleotides containing the Sp1 site. A-D) A supershift experiment was performed to assess binding of Sp1 or Sp3 in nuclear extracts from A) HT1080 cells and HeLa cells B) HUVEC and Bowes melanoma cells, to the double-stranded IRD700-labeled unmethylated oligonucleotide GCCAC**CG**ACCCCACCCCCTGCCTGGA, which includes the Sp1 site at +62 (underlined) immediately preceded by one CpG (in bold). The analysis was done for cells cultured for 24h in culture medium in the absence (-) or presence (+) of 20 nM PMA. Note that the loss of several shifted band in the presence of a large excess (10–5 M) of unlabeled competitor oligonucleotide (UC). The solid arrows indicate the supershift band obtained with antibodies to Sp1 (A and B) or Sp3 (C and D). The dotted arrows indicate the bands whose intensities are reduced by incubation with antibodies to Sp1 or Sp3.
(TIFF)
