# Supplementary figures and images for: Effect of Regulatory Element DNA Methylation on Tissue-Type Plasminogen Activator Gene Expression
Source: PLoS One. 2016 Dec 14;11(12):e0167588. doi: 10.1371/journal.pone.0167588 (PMC5156355; doi:10.1371/journal.pone.0167588)

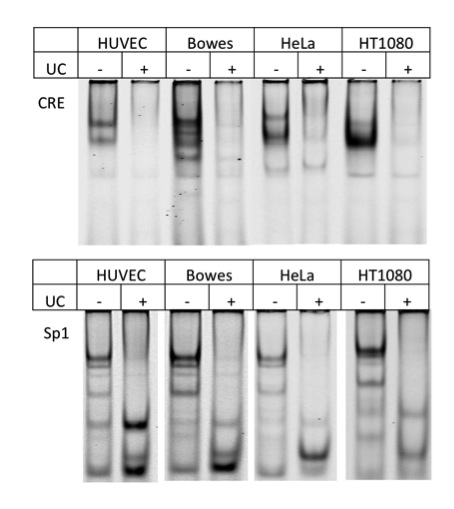

Supplement: S1 Fig — Top: Effect of an excess (10−5 M) of unlabeled double-stranded CRE oligonucleotide on the binding of transcription factors to the double-stranded IRD700-labeled unmethylated oligonucleotide TTCCTGCGATTCAATGACATCACGGCTGTG, which includes the CRE site (underlined) flanked by two CpGs (in bold). Note the reduction in intensity of the principal shifted bands. Bottom: Effect of an excess (10–5 M) of unlabeled double-stranded oligonucleotide containing the Sp1 site on the binding of transcription factors to the double-stranded IRD700-labeled unmethylated oligonucleotide GCCACCGACCCCACCCCCTGCCTGGA, which includes the Sp1 site at +62 (underlined) immediately preceded by one CpG (in bold). Note the reduction in intensity of the four upper bands. (TIFF) [file pone.0167588.s001.tiff]

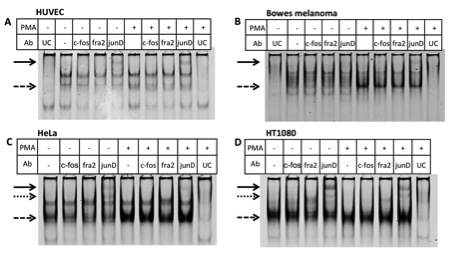

Supplement: S2 Fig — A-D) A supershift experiment was performed to assess binding of c-fos, fra2 and junD in nuclear extracts from HUVEC (A), Bowes melanoma cells (B), HeLa cells (C) and HT1080 cells (D) to the doublestranded IRD700-labeled unmethylated oligonucleotide TCCTGCGATTCAATGACATCACGGCTGTG, which includes the CRE site (underlined) flanked by two CpGs (in bold). The analysis was done for cells cultured for 24h in culture medium in the absence (-) or presence (+) of 20 nM PMA. Note that in the presence of a large excess (10–5 M) of unlabeled competitor oligonucleotide (UC) almost no shifted bands were observed. The solid arrow and the dotted arrow indicate the supershift bands obtained with antibodies to junD. The dashed arrow shows the position of a band whose intensity increases with PMA treatment of Bowes melanoma cells or HeLa cells. (TIFF) [file pone.0167588.s002.tiff]
